# Collectanea

**Published:** 1829-07

**Authors:** 


### COLLECTANEA.

Floriferis ut apes in saltibus omnia libant,

Omnia nos, itidem, depascimur aurea dicta.

### PATHOLOGY.

*Occasional Fallacy of the Pulse in Hectic Fever.*—Dr. Heberden observes, "that there are not many diseases in which an attention to the pulse affords more instruction than it does in hectic fever. Yet, even here, whoever relies too confidently upon the state of the pulse will in some cases find himself misled; for it happens, as well as I can guess, to one among twenty hectic patients, that while all the powers of life are declining, with every sign of an incurable mischief, the artery will, to the last minute, continue to beat as quietly and as regularly as it ought to do in perfect health."—*Commentaries*, p. 191.

*Case of Suppression of Semen, from Inflammation of the Prostate and Vasa Deferentia.* By Dr. Schutte.

This disease occurred in a labouring man, who had worked in a marsh for two days, standing in water up to his knees with naked limbs. He was married; and, though he had natural erections, and coition was attended by the usual sensations of pleasure, no emission took place. On examination by the rectum, the prostate was found tumid, and the surrounding parts inflamed. He was kept upon light diet, and requested to abstain from sexual intercourse. Antiphlogistic means were also employed. Leeches were applied to the perineum; and citrine ointment, mingled with sal ammoniac and extract of cicuta, were afterwards rubbed upon the same place. In five weeks the prostate had nearly regained its natural size, but still no discharge followed connexion. Dr. Schütte then ordered an injection of tartar emetic, which was persisted in for eleven days. This immediately caused great pain and an excessive discharge of mucus. On the seventh day from its application, the discharge of semen *in coitu* was perfectly natural. An infusion of bitters, and a solution of sal ammoniac, were given, to strengthen the stomach and remove the remaining enlargement of the prostate. The patient was entirely restored to health.—*Graefe und Walther's Journal der Chirurgie.*

*Remarkable Case of Abdominal Dropsy.* By J. M. Bardsley, m.d. &c.

Mrs. B., sixty years of age, was seized with swelling of the abdomen after exposure to wet and cold. The belly rapidly increased in size, and tapping became necessary. General health unimpaired. Thirst rather urgent. No cough.

1772.—The first operation was performed February 26th, and twenty-six pints of pale-coloured fluid were removed. In March, April, and May, fifty-two pints and a half were drawn off, at three different times. No further collection took place until December.

1774.—She was tapped on January 13th, and thirty-six pints were removed. In each month of this year the operation was repeated, and 312 pints were removed.

1775.—Tapped fourteen times: twice in January, and twice in December; once in each of the other months: 370½ pints were drawn off in the course of this year.

1776.—Twice in July and December, and once each other month; 352½ pints being the quantity of fluid drawn off.

1777.—Twice in August, and once each month until that time; amount this year, 244 pints. The patient died in September of dysentery.

*Remarks.*—This case appears to me to possess considerable interest. It affords proof of the enormous quantity of fluid that may be formed at successive intervals in the abdomen, without occasioning any material derangement of the general health; for the only inconvenience experienced by this lady was the excessive weight she had to support previously to each operation. Several cases are on record in which great and frequent accumulations of fluid have taken place in the abdomen; but I do not recollect more than two or three where the operation of tapping was performed fifty-three times, and 1394 pints* were removed from the same individual. Another circumstance worthy of notice is the impunity with which so frequent a repetition of the operation was borne, affording proof of the little danger or inconvenience from the practice of tapping, when properly performed. Some practitioners seem to view this operation with a certain degree of alarm, and thus do not resort to it so soon as in most instances is desirable. I have had many opportunities of witnessing the bad effects of unnecessary delay in recommending *paracentesis abdominis*, as well as the great benefits attending an early evacuation of the abdominal fluid. When the ordinary remedies in ascites have been fairly tried during a moderate length of time, and the accumulation of fluid increases rather than decreases under their use, it is of the first importance to direct immediate tapping of the belly, as affording the patient the only chance of cure. Dr. Fothergill has energetically given the same advice. In itself the operation is extremely simple, and it has been shown that, under ordinary circumstances, little or no danger attends it. "Paracentesis," as Mr. S. Cooper has judiciously remarked, "only becomes a serious measure when the disease has existed for a great length of time, and the patient has been much weakened by it."—*Edinb. Med. and Surg. Journal*, April 1829.

---

*Case of Anæsthesia, or Loss of Sensation, unattended with corresponding Loss of Motion.* By A. Reid, Esq. Surgeon. (Communicated by Mr. Liston.)

Mr. Walker, æt. fifty-six, tall and stout, and walks very erect. He is a person of humour and wit, and a good mimic. Mental powers but little impaired. Disposition perhaps rather more irritable than formerly. It was during his residence in Jamaica that the disorder first attacked him. About 1802, he fell from his horse, fractured some ribs, and injured the sternum. In consequence, he was confined to his back for several weeks, and for some time after that he was not able to walk. He gradually recovered, although he felt at this time a numbness commence in the right hip, extending to the toe. Two years after, in leaping, he sprained the muscles of the back situated over the lumbar vertebræ. He could not move without severe pain in the part. Upon his recovery, he found the numbness of his right leg rather increased, although he was still able to transact his business. No change took

* The quantity drawn off in this case.

place till 1812: he was then attacked with erysipelas in the right leg and foot, from cold. The left leg was afterwards affected. Both legs were now benumbed and insensible to the prick of a pin. His left foot was the weaker of the two. His limbs swelled occasionally, and were covered with a disagreeable eruption. In the warm bath he knew not whether the water was hot or cold, until immersed above the middle of the thigh, even although his feet and legs were in this condition. "He felt as if covered with a stocking or boot, or as if sleeping." He could still take proper exercise.

In 1815, he tried a sea voyage, and a residence in his native country. He arrived in Scotland in July. His mind and body were vigorous. The want of feeling was still increasing throughout the body. He now returned to Jamaica, having taken medical advice in London and Edinburgh without benefit. Upon his arrival, he thought himself rather better.

This amendment was not permanent. He found the heat of the sun insupportable; quite different from what he formerly felt it. He now unfortunately bruised his foot, and injured the metatarsal bone of the little toe, by which a troublesome sore was caused. His health he thought suffered from want of exercise, which he was not able to take regularly, on account of this accident.

He returned in 1818 to Scotland, worse than when he left it. In 1822, it was found necessary to remove the metatarsal bone,* which was carious, and produced great constitutional irritation. Mr. W. felt not the smallest pain during the operation.

The sentient power was now nearly, if not completely, annihilated over the whole surface of the body. Power of motion impaired, but he can carve his food, write, &c. He can also walk a short way, even without a staff. He says, "The want of feeling continued to increase slowly, and from my legs extended to my hands and arms, till I lost the feeling of finger after finger. The skin of my brow and head is also affected. I feel with nothing but my mouth: i. e. I am incapable of telling whether any thing I touch is cold or hot, rough or smooth. I am, generally speaking, in possession of my ordinary functions. With regard to the sensasion of my feet and hands, (and these I am at a loss to describe,) when cold, which they generally are, they feel heavy and stiff. When attacked with rheumatism, or when blistered from going incautiously too near the fire, (an accident of which I am not conscious at the time,) or when matter is gathering, they feel as if tight bound in a boot, and very heavy, accompanied with restlessness and stretching all over the body. This was exactly the sensation produced by the evacuation of matter which so often took place from the diseased bone. I felt no pain whatever when you extracted the bone from my foot, nor would I now, I am convinced, were you to dissect the whole foot. When driving or riding, I cannot tell, unless I see, whether or not I hold the reins or whip. My taste, smell, and hearing, are perfectly entire. Sight weak. My feet and hands are to a certain extent paralysed; that is to say, I have not the same power of motion in them which I had in a state of health, nor even a few years ago, when the want of feeling was nearly as great as it is at present."

Mr. W. is a living instance of abolition of sentient power, not only in the skin, but also in the deep-seated muscles, tendons, and ligaments, as was shown in the operation; while the power of the nerves of the other external senses remain entire, and perform their functions in a perfect manner. The

* The bone is in Mr. Liston's museum.

internal functions obey the will, and each acts correctly under the influence of their peculiar stimulus, as in a state of health. Even the *sensorium commune* is able to carry on its intellectual operations. The motive power, which at first was little affected, appears now to be getting involved in the wreck of the sentient. The extensor muscles appear more paralysed than the flexors. The reason of this is, from greater extent of muscular power being required for flexion than extension, the muscles are stronger, or additional ones are given for flexion. No more is needed for extension than almost to balance the power of the flexors.

This case confirms the physiological and pathological doctrines of Mr. C. Bell and others, relative to the double functions of the *medulla spinalis* and brain. The paralytic affection is not now confined to the lower extremities. The trunk, neck, face, and head are affected, showing distinctly that the brain has participated in the diseased structure of the spine, which was certainly injured at the time the first accident took place. It is remarkable that a diseased brain should be found capable of carrying on its intellectual operations so very entire. There are, however, some well-authenticated cases on record where the structure of the brain was diseased, and where a great part of it had been removed by absorption, leaving nothing but a cavity, yet without impairing the mental faculties.

The gentleman, whose case is above described, is still living, and can even enjoy life.—*Ibid.* (Condensed.)

---

### PRACTICAL MEDICINE.

*Singular Treatment of Traumatic Tetanus.*—We take the following extract from a review of Dr. W. Reid's work on Tetanus and Hydrophobia, in the *American Journal of the Medical Sciences.* We were not ourselves aware of the fact stated :

" It may be proper, as a matter of curiosity, merely to allude to an extraordinary practice among the inhabitants of the Tonga, or Friendly Islands, in the South Pacific Ocean, among whom we are told traumatic tetanus prevails to a great extent. It consists in producing a considerable degree of irritation in the urethra, and a discharge of blood from that part by the introduction of a reed of proper size for some distance into the canal; and, when the case is very violent, by passing a cord along the urethra through the perineum, the two ends of which are occasionally pulled to and fro, inducing great pain and a copious hemorrhage, with much swelling and inflammation of the penis. By Mr. Mariner, from whom we derive the account of this strange and unpromising practice, it is stated that he witnessed two cures of confirmed tetanus from it. Every fact relating to the treatment of this disease is interesting, and, without advising this precise mode, it may suggest a principle capable of improvement. It was, indeed, somewhat on this principle that Dr. Brown, of Lexington, many years ago, proposed exciting strangury as a cure, and bore some evidence to its efficacy."

---

*Effects of Blisters.*—Dr. Heberden* observes, that it is one among the many instances of our imperfect knowledge of the animal economy, that we can by no means understand how the cantharides should pass so quietly without

* Commentaries, p. 416.

hurting the various passages, and some of them of exquisite fineness, through which they are carried to the bladder, and yet irritate this part in that extraordinary manner which is too often experienced from the application of blisters. The difficulty of accounting for the above fact is increased by our finding that one blister has sometimes occasioned this irritation, though afterwards, in the same person and the same illness, five blisters applied at once have had no such effect; and what is called a perpetual blister, after it has been kept open seven years without at all affecting the bladder, has all at once, and without apparent reason, acted upon it so powerfully as to render the removal of the blister absolutely necessary.*

---

*Chorea.*—Dr. Harrower has recently detailed a case of this generally obstinate disease, in the Glasgow Medical Journal, which was cured by the application of the tartar-emetic ointment so as to produce pustules on the scalp, and afterwards keeping up the discharge for a short time.

---

*Upon the Application of Lunar Caustic in the early Stage of Angina.* By M. Toirac. (*La Clinique.*)

Cauterization by nitrate of silver, at the commencement of angina tonsillaris, almost always, it is said, arrests the disease. M. T. relates the following cases from his extensive experience upon this subject:

Case I.—For several years M. Toirac himself had been liable to pain in the throat, of uncertain duration, which generally arose from cold, or some deviation from his ordinary habits. Rest, mild diet, and sometimes, but very rarely, leeches, were sufficient to remedy this complaint. But the disposition which always remained to a recurrence of the disease subjected M. T. to many and tiresome precautions to prevent such repeated attacks. He determined to break through the morbid disposition of the parts; and for this purpose he had recourse to the caustic, which surpassed his expectations. When he first applied it, the right tonsil was tumefied, the palate was red and granulated. The velum pendulum palati presented the same appearances. Deglutition was difficult, and the uvula somewhat elongated, and marked with red streaks. The tongue was depressed, and each part which had the most florid appearance, and which was the most painful, was touched with the lunar caustic. In one hour every disagreeable sensation subsided.

Since this experiment M. T. has always had recourse to the same treatment in similar cases, and it has invariably been successful. The application of the caustic to the mucous membrane lining the mouth gives no pain, although the copperish taste which follows is rather disagreeable, and sometimes produces nausea. The apprehension of increasing the inflammation by this mode of treatment, is entirely chimerical.† The same observation, we are assured by M. T., will apply to cauterization by a hot iron. "I daily have recourse to it (*le fer rouge,*) for some particular affections of the gums: its application to the affected part produces scarcely any unpleasant sensation. Sometimes,

* Richerand, Physiologie, tome i. p. 43.

† In confirmation of this statement, we refer to the experience of Mr. Higginbottom, of whose work upon the use of Lunar Caustic in various inflammatory diseases a review is given at page 521 of our last Number. —Editors.

indeed, the patient is almost unconscious of it, and is only aware of the operation from the hissing which results from the contact of a heated iron to a humid part!"

Case II.—Mademoiselle A. G. had been frequently subject to sore throat. The same treatment was adopted with similar advantage. The tonsils were previously much enlarged, but afterwards they presented nearly a healthy appearance. Whenever she now experiences any precursory symptoms of her former malady, she has recourse to the argentum nitratum, and in a few minutes she is relieved from all inconvenience. It is proper to observe that this patient had frequently been submitted to the ordinary modes of treatment. Her neck was covered with numerous leech-bites. The slightest exposure to cold, particularly if her neck was uncovered, had always caused an attack of sore throat, to which she was, after the above treatment, not more disposed than the generality of people.

Case III.—A young lady had, from five years of age, been frequently subject to affections of the throat. Her tonsils had sometimes been so much enlarged as to threaten suffocation. By the application of the Nitr. Argent. the malady disappeared, as if by enchantment.

M. T. refrains from adding many similar cases in corroboration of the efficacy of the treatment he proposes. We are not to imagine that bleeding, and other remedial means, may not be requisite in some cases.

### SURGERY.

*Nævus Maternus cured by Vaccination.*—Catherine Strathern, eight months old, was brought to the Glasgow Royal Infirmary in the month of September, having a nævus on the lower part of the forehead, half an inch above the left inner canthus. It was as large as a hazel nut, and of a dark red colour. It was observed at birth, and was then quite level with the surface. After a month it became elevated. Having never been vaccinated, fresh lymph was inserted by minute punctures, both around the circumference and over the whole extent of the tumor. On the eighth day many small pustules were visible, and by the twelfth they had coalesced and become incrusted. On the twenty-first the scab separated, leaving the surface underneath tender and slightly prominent. A second crust succeeded, and to this a third and a fourth; a perfect cure being effected in about six weeks.

The narrator of this case is of opinion, that it is indispensable to the ultimate success of the practice that the lymph should be freely introduced over the diseased surface, as well as around its circumference. In this way the adhesive inflammation which is excited appears to extend from one pustule to another, and in the course of a few days the whole becomes involved in one scab.

*Case of Tracheotomy.* By Zadok Howe, M.D. of Billerica, Massachusetts.

A daughter of Mr. French, of Tewksbury, three years and a half old, on the 21st of September last, while at play in the garden, took a bean into the trachea.

I saw the child two hours after; but, as we had no means of ascertaining the nature of the foreign substance, and as the symptoms were not very urgent at the time, I heard nothing more of the case till the 28th. The child was then labouring under frequent and distressing paroxysms of coughing, attended with suspended respiration and other urgent symptoms, which clearly indicated the necessity of an operation. It was proposed, and immediately assented to by the parents. It was evening; and, as I was unprepared for an operation, it was postponed till the next day, when, with the assistance of Dr. Dalton, of Chelmsford, it was performed in the following manner :

A heavy table was provided, with the side leaves turned down, leaving a horizontal surface, sixteen inches wide, covered with blankets, with a firm roll of cloth four inches in diameter across the end.

The child was firmly secured on the back by the hands of assistants, the nape of the neck resting on the roll of cloth, the head carried far back over the end of the table. An incision was made from the lower edge of the thyroid cartilage to within a quarter of an inch of the sternum. After waiting a few moments for a slight bleeding to subside, a puncture was made into the trachea with a slender double-edged scalpel, in the centre of the incision, dividing one cartilage; then, with a curved, probe-pointed bistoury, the puncture was dilated from within outwards, dividing one cartilage above and one below. In this elongated state of the parts, the division of three cartilages made an opening sufficiently free to admit the forefinger of the left hand into the trachea. The finger was introduced to separate the edges of the incision which did not incline to retract. Immediately after withdrawing the finger, with a spasmodic effort, a bean was expelled with considerable force, and lodged on a bed which stood in the room. This saved us the trouble of attempting that part of the operation which I most dreaded; for experience had taught me to envy no man the pleasure of probing in the trachea for beans or peas. Half an hour after, the opening still retained the shape of the finger, large and free: the divided cartilages had approximated but very little. The wound was then brought together, and secured with adhesive plaster; and, being unwilling to disturb the stomach, we gave no medicine, excepting a few drops of laudanum, at the same time directing a spare diet. The plasters succeeded imperfectly, partly in consequence of the action of the mastoid muscles, and because the opening was rather too low on the neck to admit of their being applied to the best advantage. The air rushed through the aperture occasionally for forty-eight hours, but never after.

I dressed the wound a few times, and discontinued my attendance in about two weeks. At the time of the accident, the child had not entirely recovered from the hooping-cough, but the cough troubled it very little after the operation. The wound was cicatrised at the end of eighteen days. A short time previous to this, a slight dysenteric affection took place, for which the family gave some domestic medicines. A few worms were discharged, and the child soon recovered, the cough wholly subsiding at about the same time.

The result of this case may, I think, be attributed in part to the position of the child when the opening was made. By carrying the head very far back over the cylinder of cloth, the trachea became considerably curved.

In the act of coughing, the bean was suddenly carried from one end of the trachea to the other, and, when forcibly propelled, would probably incline to the longest side of the curved tube: the opening being in that part, and as large as the cavity of the trachea, we had some reason to expect what actually took place, the expulsion of the bean. By introducing the finger, and turning it a quarter round, the elasticity of the cartilages seemed to be destroyed, or at least suspended for a length of time sufficient for our purpose. In an older subject the elasticity might not have been so easily overcome in this manner. The operation never seemed much to affect the general health of the child; and the most difficult part of the after-treatment was to restrain the immoderate indulgence of the appetite for food.—*American Journal of the Medical Sciences.*

---

*Re-union of a large Portion of the Calf of the Leg, which had been torn off by accident.*—It occurred in the practice of Dr. GRÖSCHNER, of Spremberg. A labourer was employed with a companion in carrying timbers for a building. While he was engaged in moving a beam, carrying one end of it, having both hands behind him, and sustaining the load about as high as his loins, another piece of timber fell across that they were carrying, and struck the end violently from his grasp. The sharp corner of the beam struck him about a hand's breadth below the bend of the knee, and tore down the integument and the gastrocnemius internus muscle, nearly to the tendo-achillis. The breadth of the flap above was upwards of three inches; below, it was held to the limb by scarcely a finger's breadth of integument alone. The persons who conveyed the man to his house had re-applied the flap of flesh, but, to check the bleeding, had washed the limb with brandy, and wrapped it in cloths wet with the same. Dr. Gröschner removed these, and applied adhesive straps. The patient did well for a few days, but soon became very low, and at length had regular hectic and night sweats. Finding little prospect of adhesion, Dr. G. made use of sutures, and a wash to stimulate the integument to a higher degree of action, administering to the patient at the same time Peruvian bark in considerable quantity, and a nutritious diet. To his great satisfaction, adhesive inflammation took place, and the whole mass became firmly united.

If the violence of the shock to the limb be recollected, and the fact that the mass of the gastrocnemius detached was to derive its circulation, exclusively, through little more than half an inch of integument at the inferior part of a lower extremity, we may regard this cure as a triumph of considerable magnitude. The patient was able to resume his business on the twenty-ninth day after the accident, notwithstanding all the fluctuations of health he had undergone in that time.—*Græfe und Walther, Journal der Chirurgie und Augenheilkunde.*

---

*On the Cicatrization of the Womb after the Cæsarian Section had been successfully performed;* by Professor MAYER, *of Berlin.*

The cicatrization of the womb, Dr. M. remarks, has rarely been examined after the cæsarean section had been successfully performed. He therefore describes the condition of a preparation added to the anatomical cabinet of Bonn, taken from the body of a woman, who, eight years previous to her death, had successfully endured the cæsarean operation, by which the life of herself and child had been preserved.

The operation was performed by HOFRATH VELTEN, in March 1813, who furnished the history of the case. The distance from the under edge of the symphisis pubis to the promontory of the sacrum was scarcely two French inches; the patient was thirty-six years old. The abdomen was opened in the linea alba, and the incision through the womb extended five inches downwards. During the division of its parietes, the womb did not contract in the slightest degree. The infant, a stout boy, and the placenta, were removed from the womb, without any remarkable hemorrhage ensuing; perhaps not more than a pound altogether. From the difficulty of bringing the now greatly relaxed abdominal parietes together, it was necessary to resort to stitches, and this is said to have been the most painful part of the operation. The patient, when questioned after her recovery as to her feelings during the operation, stated that the sensation caused by the incision through the abdomen she could only compare to what would be produced by drawing a redhot needle over her skin. She assured us that the incision through the womb itself did not cause her the slightest degree of pain.* In the month of May, three months after the operation, the wounds were entirely healed, and the mother and child enjoyed the best health.

Eight years after, she died, and the uterus was examined by Professor Mayer, who found it in the following condition:

The uterus had perfectly regained its natural form and consistence. The length of the womb, from the superior part of the fundus to the edge of the anterior lip of the os uteri, was two inches, seven lines (French) long. The diameter, from the insertion of one fallopian tube to the other, one inch, ten lines. On the external surface of the anterior wall of the uterus, the place of the incision through it was indicated by a furrow about one fourth of an inch long. The peritoneum adhered firmly to the substance of the womb at this point, and covered the furrow above mentioned. The edges of the wound through the uterus were extremely contracted and drawn inwards; the cicatrix on the inner surface was two lines and a half long. It here projected into the cervix uteri, and commenced by a depression a line and a half broad. The anterior wall of the womb in the vicinity of the cicatrix was three lines thick; the posterior, opposite thereto, four. The mouth of the uterus was natural; but a long, thin, fleshy polypus descended from the cervix. The fallopian tube and ovary of the left side were natural; on the right side, the tube and ovary were grown together. Several cicatrices appeared on the ovaries.

From the foregoing description, it appears that, as no new-formed mass occupied the place of the wound made in the uterus by the cæsarean section, but the edges were directly united, the union took place either by the first intention, or the intermediate substance (if such had existed) was subsequently absorbed.—*Ibid.*

---

*Remedy for Opacity of the Cornea.*—The following combination is recommended in the *Journal de Chimie Medicale, &c.* for September 1828, as an useful application to promote the absorption of the effused lymph in opacity of the cornea.

℞. Oxyd. Hydr. rub., Agaric alb. āā ʒss.; Sacch. alb. ℥i. Misce intim. et pulv. subtil. A small portion to be blown into the eye daily.

---

*Lithotomy.*—Of eighty-three operations by the lateral method, performed by M. J. M. Viricel, at the Hôtel Dieu of Lyons, eighty were successful.—*Revue Médicale.*

* "Beim Einschnitte in den Uterus selbst gab sie uns die Versicherung, dass sie davon nicht den mindeste Schmerz empfunden habe!"

### MIDWIFERY.

***Extra-uterine Fœtation.*—Ellen Bryan, a married woman, aged thirty years, became pregnant in the year 1821. At the period of her expected parturition, she felt something break off from its place inwardly, and she was visited by her medical attendant. But the time passed away without any delivery, and without any diminution of her abdominal bulk. A new state of gestation supervened in a year and a half afterwards, and terminated, at the end of nine months, in the birth of a full-grown female infant. At the expiration of about two years more, Bryan gave premature birth to a live babe, which seemed to have attained only the close of the sixth month. She again, for the fourth time, became pregnant in 1826, and was, after seven months' gestation, delivered in Cork-street Hospital of a child, which, like the preceding one, survived its birth only a few hours. The original tumor remained unabated.

I first saw this poor woman upon her admission into one of my wards, on the 19th of May, 1827. The following state of disease presented itself to my observation: Countenance pale, dejected, and expressive of long suffering; eyes sunken; much general emaciation, pulse slightly accelerated, and extremely weak; abdomen of immense bulk and prominence, palpably enclosing a large solid substance like a fœtus; the centre of this prominency pointing forwards, and exhibiting an incrusted ulcer on its apex, a little below and to the right side of the umbilicus. Diarrhœa.

21st May.—The incrustation at the apex of the above-mentioned prominency has detached itself and fallen off, and has left an aperture into the abdominal cavity, from which there is a discharge of much fetid matter, partly ichorous and partly purulent.

27th.—Diarrhœa unabated; abdominal pain not acute, except when it is rendered so by pressure, or by an unfavorable position of the patient; a most offensive odour emitted from the open abdominal ulcer; a gradually increasing dilatation of this aperture; a feeling on the part of the patient of being hurt interiorly by a bone. She takes a night pill containing one grain of opium; and, with a view of obviating as much as possible her hourly increasing debility, I direct for her four grains of sulphate of quinine twice a day, and twelve ounces of port wine, which she likes much.

9th June.—The abdominal aperture was hourly becoming larger. It was so wide yesterday as to expose to view the entire cause of this poor woman's tedious malady: viz. the inanimate body of a full-grown male infant, which lay out of the womb in the abdominal cavity during more than the preceding six years. It has been taken out by our very intelligent surgeon, Mr. Trant, and is preserved in spirits of wine. It is covered with a semi-coriaceous cutis, that is particularly distinguished over almost the whole of the surface of the shoulders, back, and seat, by an adipocire-like appearance. Our poor patient is barely alive. Her pulse is scarcely perceptible. Portions of intestine have been destroyed by abrasion and sphacelation, and the feces pass out anteriorly through the opening.

13th.—She has ceased to exist.

14th.—Of the appearances exposed to view by the anatomical examination of the body, the following are the principal: An ample cyst, containing much pus, is situated behind and a little above the right side of the fundus uteri. The thick envelope of this sac consists in a prolongation of the peritoneum, especially of the right ligamentum latum. The right fallopian tube is distinctly traceable from both ends towards its centre, where it is connected with the external side of the parietes of the cyst, and is there lost in consequence of its being confounded with this envelope. The right ovarium is somewhat flattened on its posterior side, where it is close to the adjoining side of the cyst. The left ovarium, and corresponding fallopian tube, are sound, and in their natural situations. The uterus is sound, and does not bear the slightest vestige of any former laceration or of any kind of disease.—*Appendix to* Dr. O'Reardon's *Report of the Dublin Fever Hospital.*

### NATURAL HISTORY.

*Instinct of Spiders.*—A small spider (*Epeira diadema,* Latreill,) had spread its net between two neighbouring trees, at the height of about nine feet. The three principal points to which the supporting threads were attached, formed here, as they usually do, an equilateral triangle. One thread was attached above to each of the trees, and the web hung from the middle of it. To procure a third point of attachment, the spider had suspended a small stone to one end of a thread; and the stone, being heavier than the spider itself, served in place of the lower fixed point, and held the web extended. The little pebble was five feet from the earth. The whole was observed, and is described, by Professor Weber, of Leipsig.—*Archive für Anatomie.*

*On the Sexual Instinct of Insects.* By J. H. Davies.—It has been asserted that the circuitous flight of the butterfly tribe arises from one sex pursuing through the air the track of the other; and that, if an unimpregnated female of the *Phalæna quetcus* (egger moth) be carried in a gauze cage into the haunts of that species, numbers of the males will be attracted, so as to be easily captured. I have never had an opportunity of verifying this fact; but, from a circumstance which occurred to me during the past year, I have no doubt of its correctness.

I was engaged in rearing lepidopterous insects from the larvæ, and had a great variety of the pupæ of different species. One evening I found a female *Sphinx ocellata* just emerged, which, in lifting from the floor, ran up my arm and round the collar of my coat: two hours after, on returning to my study from shutting some glass frames in the garden, a very fine male, of the same species, was fluttering on my shoulder, where the female had previously crawled. But a still more curious fact, which must appear almost incredible, remains to be stated. Two females of the *Sphinx populi* were evolved. The next day I found three males in the room. As no one had entered it in the interval, nor was there apparently any mode by which they could gain access, I was somewhat puzzled to account for their appearance. The same evening, however, the mode of entrance was made apparent, by two more males, of the same species, coming down the chimney; one of which fell into a vase standing on the fire-place, where I captured it before it could extricate itself. Afterwards, upon occasion of the evolution from the pupa state of females of the *Phalæna bucephala* and *Phalæna salicis,* the windows of my study were completely besieged by males of the same species, which, upon throwing open the windows, eagerly rushed in.—*Quarterly Journal of Science.* Curious indee[illegible]

### MISCELLANEOUS.

*Leech-bites.*—Dr. Löwendhart mentions a method of checking the profuse bleeding from leech-bites, which is simple and effectual. The edges of the little wounds are drawn together with a fine needle and thread. The thread, being drawn through the cuticle only, gives no pain, and the bleeding is at once suppressed.—*Græfe und Walther, Journal der Chirurgie.*

---

*Opium Eater.*—Mustapha Shatoor, an opium eater in Smyrna, took daily three drachms of crude opium. The visible effects at the time were sparkling of his eyes, and great exhilaration of his spirits. An increased dose became necessary. He seemed twenty years older than he really was. His complexion was very sallow, his legs small, gums eaten away, and the teeth laid bare to the sockets. He could not rise without first swallowing half a drachm of opium.—*Phil. Trans.* xix. 289.

---

